# Understanding lactate in the development of Hepatitis B virus-related hepatocellular carcinoma

**DOI:** 10.1186/s13027-024-00593-4

**Published:** 2024-07-15

**Authors:** Sheida Behzadi Sheikhrobat, Shahab Mahmoudvand, Salva Kazemipour-Khabbazi, Zahra Ramezannia, Hossein Bannazadeh Baghi, Somayeh Shokri

**Affiliations:** 1 Department of Virology, School of Medicine, Ahvaz Jundishapur University of Medical Sciences, Ahvaz, Iran; 2 Research Center for Molecular Medicine, Hamadan University of Medical Sciences, Hamadan, Iran; 3 Department of Virology, School of Medicine, Hamadan University of Medical Sciences, Hamadan, Iran; 4 Department of English Language and Persian Literature, School of Medicine, Hamadan University of Medical Sciences, Hamadan, Iran; 5 Department of Virology, Faculty of Medicine, Tabriz University of Medical Sciences, Tabriz, Iran; 6 Infectious and Tropical Diseases Research Center, Tabriz University of Medical Sciences, Tabriz, Iran; 7 Immunology Research Center, Tabriz University of Medical Sciences, Tabriz, Iran

**Keywords:** Hepatitis B Virus, Lactate, Hepatocellular Carcinoma, Immune System

## Abstract

Hepatitis B Virus (HBV) is a hepatotropic virus that can establish a persistent and chronic infection in humans. Chronic hepatitis B (CHB) infection is associated with an increased risk of hepatic decompensation, cirrhosis, and hepatocellular carcinoma (HCC). Lactate level, as the end product of glycolysis, plays a substantial role in metabolism beyond energy production. Emerging studies indicate that lactate is linked to patient mortality rates, and HBV increases overall glucose consumption and lactate production in hepatocytes. Excessive lactate plays a role in regulating the tumor microenvironment (TME), immune cell function, autophagy, and epigenetic reprogramming. The purpose of this review is to gather and summarize the existing knowledge of the lactate’s functions in the dysregulation of the immune system, which can play a crucial role in the development of HBV-related HCC. Therefore, it is reasonable to hypothesize that lactate with intriguing functions can be considered an immunomodulatory metabolite in immunotherapy.

## Introduction

Cell metabolism refers to the collection of chemical reactions that occur in living organisms to maintain them. Glycolysis, fatty acid synthesis (FAS), and glutaminolysis are three main metabolic-activated pathways that generate energy in the form of adenosine triphosphate (ATP). In mammalian cells, glycolysis and oxidative phosphorylation (OXPHOS) are two primary ATP-producing pathways [1]. A normal cell converts glucose to pyruvate, which is then oxidized to carbon dioxide in the tricarboxylic acid (TCA) cycle, producing the reduced electron carriers, nicotinamide adenine dinucleotide (NADH) and flavin adenine dinucleotide (FADH2). In the presence of oxygen (aerobic glycolysis), FADH2 and NADH are utilized in OXPHOS to create 36 molecules of ATP per glucose molecule [2]. In anaerobic conditions, glucose is primarily used for glycolysis, where it is metabolized to pyruvate, then converted to lactic acid (also called “lactate”), and finally pumped out of the cell [3]. In physiological conditions, extracellular lactic acid has a concentration of 1.5mM and 20–40mM in pathological conditions [4]. Lactate is now identified as a regulator of immune cell metabolism, capable of regulating the immune-inflammatory response, angiogenesis, and fibrosis [5].

As we know, lactate is the end product of anaerobic glycolysis. In addition to serving as energy production, recent investigations have revealed that lactate plays a role in immune regulation within the tumor microenvironment (TME) [6, 7]. Several viruses, including tumor viruses, can induce aerobic glycolysis and lactate production, indicating a potential role of virus-induced glycolysis in tumorigenesis [8, 9]. In this regard, viruses may hijack host metabolism in order to produce the energy and macromolecules required for replication and spread [10]. Therefore, understanding the crosstalk between cellular metabolic pathways and viruses is essential. Hepatotropic viruses are highly adapted to hepatocytes. The lactate production within liver cells by hepatitis B Virus (HBV) significantly influences antiviral immunity, persistent infection, and subsequent outcomes, thus making it an area of research interest [11]. In this review, we have described how lactate can affect the escape of HBV from the immune system, which can be investigated as a potential therapeutic target in the future.

## Lactate

Otto Warburg demonstrated in the 1920s that cultured tumor tissues have high glucose uptake rates and preferential lactate production to provide ATP, nucleotide, lipid, and amino acids, even in the presence of oxygen; this phenomenon was termed the Warburg effect [12]. Emerging studies have shown that extracellular lactate has regulatory functions in innate and adaptive immune cells and induces dramatic changes in gene expression [13, 14]. Therefore, lactate is not simply a “waste product” of glycolysis.

Cancer patients with high blood lactate dehydrogenase (LDH) levels have poor clinical results in immunotherapy, chemotherapy, and targeted therapy [15]. Hepatocellular carcinoma (HCC) can lead to acute-on-chronic liver failure (ACLF) in patients with underlying liver disease [16] and in patients with HCC, LDH levels can serve as a prognostic indicator for clinical outcomes [17]. Activation of chronic HBV infection (CHB) is a significant contributor to ACLF [18]. A study found that lactate as the main metabolite is high in ACLF patients and can be helpful in prognostic assessment and clinical decision-making in HBV-ACLF patients [19]. Yang et al. indicated that lactate is an independent risk factor for the 90-day prognosis of patients with HBV-ACLF and its addition to current models could enhance the predictive value [20]. Another study showed that the initial lactate level strongly and independently predicts long-term outcomes and mortality in patients with HBV-related decompensated cirrhosis, including end-stage liver disease (MELD) score and Child-Pugh score [21]. The prognostic value of lactate is not well established; however, as liver dysfunction is associated with higher serum lactate levels, it is believed to be a simple and accurate prognostic marker [22].

## Hepatitis B Virus (HBV)

HBV is a significant driver of liver disease, including liver inflammation, cirrhosis, and HCC. This enveloped virus has a partially double-stranded DNA genome and belongs to the *Hepadnaviridae* family within the genus *Orthohepadnavirus*. The worldwide infection of HBV is estimated to affect over 2 billion individuals. Among these, more than 350 million people are afflicted with CHB infection, a severe condition that is often associated with cirrhosis and liver cancer. HCC is one of the most common liver cancers in humans, which accounts for about 90% of primary liver cancer [23, 24]. The Warburg effect promotes the proliferation of HCC cells, tumor cell metastasis and inhibits apoptosis of HCC cells [25].

**Fig. 1:**
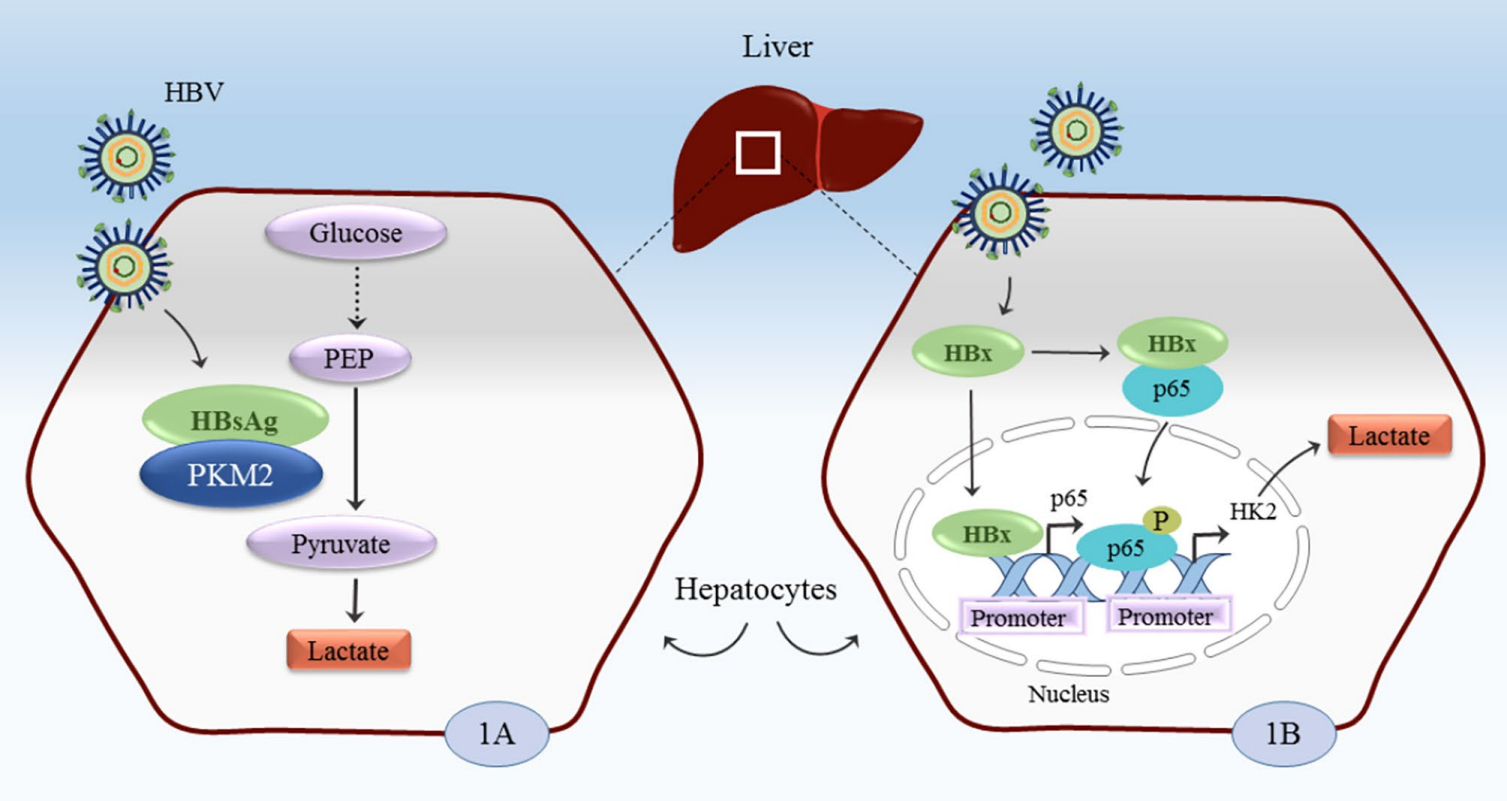
Hepatitis B virus utilizes HBx and HBsAg to produce lactate. 1**A**: pathway of HBsAg effect; 1**B**: pathway of HBx effect

Recently, the crosstalk between immunometabolism and viral infections has been considered a fascinating field among researchers. Mindful of this, it is noteworthy that the reproduction of HBV as an intracellular pathogen is contingent upon the occupation of the host metabolism machinery [26]. Hepatitis B virus X protein (HBx) is critical in hepatocarcinogenesis [27]. Mitogen-activated protein kinase (MAPK) pathways are important signaling pathways that regulate various cellular programs. Studies have shown that HBx induces the activation of MAPK pathways [27, 28] which in turn promotes the Warburg effect [1, 29]. Recently, Chen et al. have indicated that HBx induces aerobic glycolysis via the NF-κBp65/hexokinase 2 (HK2) pathway to overproduce lactate, increasing hepatocyte proliferation through PI3K (phosphatidylinositide 3-kinase)/Akt signaling and resulting in HBV-related HCC (Fig. 1B) [30]. Nuclear factor κB (NF-κB) activity controls the balance of glycolysis [31]. Pyruvate kinase M2 (PKM2) is involved in glucose metabolism, promoting the formation of the Warburg effect [32]. Wu et al. demonstrated that large viral surface antigens (LHBS) decrease PKM activity in hepatocytes, leading to increased overall glucose consumption and lactate production (Fig. 1A) [33]. Hepatitis B virus X-interacting protein (HBXIP) is a conserved protein encoded by the HBXIP gene in humans. In 1998, HBXIP was initially recognized in the HCC cell line HepG2 for its ability to interact directly with the C-terminus of HBx. By induction of PI3K/Akt and inactivating p53 signaling pathways, HBXIP can activate the pentose phosphate pathway (PPP), leading to the Warburg effect, which promotes cell proliferation, migration, and invasion [34].

### Effects of Lactate on the Immune System

The interaction between innate and adaptive immune cells leads to a highly efficient and effective immune response, and a wide range of immune cells can be found in the TME [35]. It was previously thought lactate was a metabolic waste product of cellular metabolism; nevertheless, we now know lactate plays a vital role in regulating cellular functions [36]. Several recent studies have discussed that lactate induces immunosuppression to the evasion of host immune response, resulting in chronic infections such as HBV [6, 13, 36]. Nevertheless, it is unknown the role of lactate in regulating innate and adaptive immune responses to chronic infections. Therefore, the findings of this review will provide some insight into how lactate modulates the host immune system, which will, in turn, promote the development of immunotherapeutic strategies for HBV treatment by improving the HBV-specific immune response.

#### Macrophages (MΦs) and dendritic cells (DCs)

C-C chemokine receptor type 7 (CCR7), a G-protein coupled receptor (GPCR), is essential for regulating homeostasis and disease progression. It is expressed in various peripheral immune cells, including dendritic cells (DCs) and T-cells. When CCR7 is upregulated, CCR7^+^ peripheral immune cells migrate towards lymph nodes to adjacent lymph nodes to launch the onset of adaptive immunity [37]. The presence of lactate leads to a decrease in CCR7 expression [38]. Therefore, lactate can block the maturation, differentiation, activation, and antigen presentation of peripheral immune cells such as DCs [38, 39]. Yonejima et al. demonstrated that DCs from patients with HBV displayed reductions in antigen-presenting capacity, migration capacity, and cytokine production capacity [40]. Studies have also revealed that DCs are reduced in number and impaired in function in the blood of patients with HCC [41]. The functional activity of CD8^+^ T cells against tumors is primarily attributed to the production of cytokines like interleukin-2 (IL-2), tumor necrosis factor-alpha (TNF-α), interferon-gamma (IFN-γ), and cytolytic granules such as granzyme B (GB) [42]. Based on the CCR7 expression, the analysis of CD8^+^ T cell subsets showed that the frequency of effector memory CD8^+^ T cells (TEM) of patients with advanced HCC was significantly lower than that of patients with early HCC [43].

Macrophages are classified into two different types: classically activated M1 and alternatively activated M2. M1 macrophages are pro-inflammatory macrophages activated by microbial products. They can secrete several pro-inflammatory cytokines, such as TNF-α, IL-12, IL-23, IL-1β, IL-6, and chemokines, as well as reactive oxygen species and nitric oxide (NO). On the other hand, M2 macrophages are anti-inflammatory macrophages that secrete anti-inflammatory factors such as IL-10 [44, 45]. It is now accepted that M2 macrophages have a pro-tumor role, while M1 macrophages have anti-tumor effects [46]. Based on the applied stimuli and the resultant transcriptional changes, M2 macrophages can be subdivided into M2a, M2b, M2c, and M2d [47]. This is while in the presence of lactate, macrophages are polarized towards the M2 phenotype [38]. Lactic acid-induced polarization of macrophages leads to an increase in vascular endothelial growth factor (VEGF) expression, resulting in a positive feedback loop that enhances angiogenesis [4]. Bility et al. observed an increase in M2 macrophages infiltrating the liver in CHB patients with fibrosis and/or HCC, as well as in patients with acute HBV-associated liver failure [48]. Enhanced expression of VEGF has been observed in HBV related HCC. Research studies have indicated that increased expression of VEGF serves as a valuable prognostic marker for HCC. Consequently, therapeutic interventions aimed at targeting VEGF or its receptors (VEGFR1/R2) have demonstrated significant improvements in the clinical outcome of patients with HCC [49]. In fact, HBV modulates liver macrophage functions to favor the establishment of infection [50].

#### Natural killer (NK) cells

NK cells are innate immune cells that share their role as cytotoxic and IFN-γ producing cells with CD8^+^ T cells against virus-infected cells [51]. As we know, IFN-γ plays a crucial role in modulating the immune response, particularly the host response against intracellular pathogens and tumor immunosurveillance [52]. By producing IFN-γ, NK cells can promote the maturation, effector functions, and cross-presentation of DCs, hence promoting T cell responses [53]. The nuclear factor of activated T cells (NFAT) family of transcription factors, which includes NFAT1, NFAT2, and NFAT4, plays a crucial role in gene transcription in T cell activation [54]. Lactate has been found to inhibit the activation of NFAT in NK cells, which in turn inhibits their production of IFN-γ [55, 56]. Lactate also induces apoptosis of NK cells by lowering the intracellular pH, resulting in mitochondrial dysfunction [57, 58].

NK cells represent the primary immune cell type in the liver, making up to 50% of hepatic lymphocytes [59]. NK cells play an essential role in the immune response to acute and chronic HBV infection [60]. Shi et al. found that hepatic NK cells are reduced and deactivated in HBV-infected patients [61]. The critical role of NK cells in HCC is their anti-tumor ability. They initiate T cell response through chemical signals and enhance the density of immune cells [62].

#### Neutrophils

Neutrophils are the first host defense line of the innate immune system during inflammation [63]. The neutrophil-to-lymphocyte ratio (NLR) observed in the peripheral blood serves as a prognostic biomarker. This ratio reflects the balance between systemic inflammation and immunity [64]. Increased numbers of neutrophils promote tumor growth and metastasis through angiogenesis [65, 66]. Several studies have established a higher NLR in patients with CHB [67, 68].

Over the past decades, the formation of neutrophils extracellular traps (NETs) has been associated with cancer progression, metastasis, and cancer-associated thrombosis [69]. Lactate was found to be released by human neutrophils [70], and it induces through MCT1-dependent PAD4 activation and glycolysis [71]. Guan et al. showed that the induction of NETs by HCC cells is associated with disease prognosis [72]. A recent study showed the presence of NETs in HBV-related clinical specimens and cytological analysis [73].

#### T cells

T cell, called T lymphocyte, is an essential part of the immune system. Glycolysis and lactate are crucial for T cell biology. By increasing H^+^ and maintaining a low pH in the TME, lactate suppresses the anti-tumor activity of T cells [74]. Monocarboxylate transporters (MCTs) are membrane proteins functioning as transporters for pyruvate, lactate, and ketone bodies across the cytoplasmic membrane into the intracellular or extracellular [75]. Elevated lactate blocks the MCTs of T cells. This condition causes lactate to accumulate in T cells, thus reducing glycolysis, which leads to a reduction of the intracellular phosphoenolpyruvate (PEP) level. The PEP level is a necessary glycolysis metabolite for T-cell receptor (TCR)-mediated activation [76]. In addition to NK cells, IFN-γ is produced by natural killer T (NKT) cells, CD8^+^ T cells, and CD4^+^ T cells. Lactate negatively correlates with the survival and activation of T cells. Therefore, by inhibiting TCR, lactate can impair the functions of T cells (proliferation, function, and movement), which are essential for IFN- γ production [6, 77–79]. The nicotinamide adenine dinucleotide (NAD^+^) is a critical metabolic intermediate that plays an essential role in many enzymatic reactions of energy metabolism. Many enzymes, such as sirtuins, adenosine diphosphate (ADP), ribose transferases, and synthases, utilize it as a co-substrate [80]. As NAD^+^ is crucial for the expression of the focal adhesion kinase (FAK) family interacting protein of 200 kDa (FIP 200), lactate induces T-cell apoptosis by reducing the levels of NAD^+^. Thus, lactate inhibits effector T cells and can blunt tumor immunosurveillance by T cells [81–83]. HBx protein with increased lactate production impairs effector T cell function in the liver [84].

NKT cells act as a bridge between innate and adaptive immunity. IFN-γ production is necessary for the anti-tumor effects of NKT cells [85]. Low extracellular pH inhibits NKT cell functions by inhibiting mammalian target of rapamycin (mTOR) signaling and nuclear translocation of promyelocytic leukemia zinc-finger (PLZF) [86]. The peroxisome proliferator-activated receptors (PPARs) are a group of nuclear receptor proteins that regulate physiological events of inflammation and metabolism. The PPAR superfamily consists of three isoforms, PPARα, PPARγ, and PPARβ/δ, with different tissue distributions [87, 88]. PPARγ, a regulator of lipid metabolism, promotes activation and IFN-γ production in iNKT cells. Lactate can repress the anti-tumor activities of NKT cells by reducing the PPARγ [89]. In HBV infection, NKT cells play important roles in early infection control [84].

The regulatory T cells (Tregs), as suppressor T cells, that modulate the immune system represent a unique subpopulation of T-helper 1 (Th1). The suppressive activity of Treg involves the coordinate activation of several genes. This activation, including Cytotoxic T-Lymphocyte Associated Protein 4 (CTLA4) and TNF Receptor Superfamily Member 18 (TNFRSF18) by forkhead box P3 (FOXP3) along with repression of genes encoding cytokines such as IL-2 and IFN-γ. Lactate inhibits T cell glycolysis, which leads to FoxP3 expression and promotes Treg differentiation [90].

Elkhal et al. demonstrated that NAD^+^ promotes the conversion of Tregs into Th17 cells [91]. On the other hand, by inhibiting glyceraldehyde-3-phosphate dehydrogenase (GAPDH) binding to the AREs of the IFN-γ mRNA, NAD^+^ up-regulates IFN-γ expression. By decreasing the levels of NAD^+^, lactate reduced the expression and secretion of IL-17 A cytokine and IFN-γ production. [91, 92]. Several studies have shown that during chronic HBV infection, Treg cells proliferate dramatically and suppress the anti-HBV immune response [93, 94].

#### RLR signaling

**Fig. 2:**
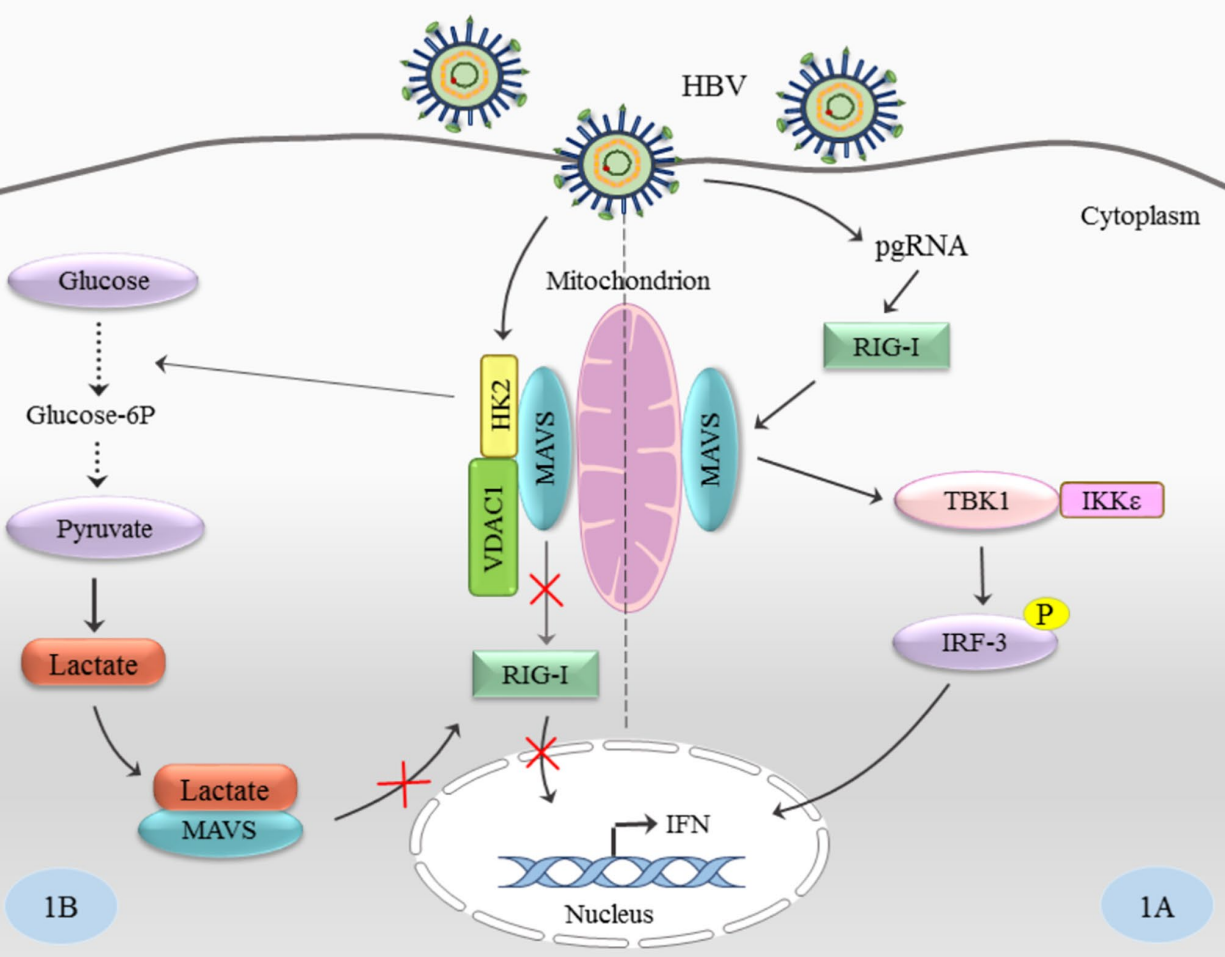
The role of HBV in the inhibition of RLR signaling through the HK and lactate. **1 A**: The usual pathway of the host innate immune response; **1B**: Suppression of the RLR signaling by HBV

Retinoic acid-inducible gene I (RIG-I)-like receptors (RLRs) have a crucial role in recognizing viral RNA in the cytosol and then mediating the transcriptional induction of type I interferons (IFNs-I). This process leads to an effective antiviral response [95]. RLRs interact with the N-terminal caspase activation and recruitment domain (CARD), found in mitochondrial antiviral-signaling protein (MAVS). This domain functions as the essential adaptor protein for RLR signal transduction. Activated MAVS recruits TANK-binding kinase 1 (TBK1) and IκB kinase-ε (IKKε), which subsequently activate interferon regulatory factor 3 (IRF3) and IRF7; these, together with nuclear factor-κB (NF-κB), then induce transcription of the genes encoding IFNs-I and other antiviral or immunoregulatory genes [95]. HBV can affect and increase glucose metabolism. In fact, HBV leads to lactate production by increasing HK activity and stimulating LDHA [78, 96]. HBV induces the formation of a trimeric complex between HK2, MAVS, and VDAC1, resulting in increased interaction between lactate and MAVS. Therefore, lactate suppresses RLR signaling via binding with MAVS, subsequently inhibits the RIG-I/MAVS interaction, and blocks IFN production (Fig. 2) [97, 98].

### Effects of lactate on epigenetic regulation

Epigenetic changes are genetic modifications that impact gene activity without modifying the DNA sequence [99]. Histone lysine lactylation (Kla) is a new epigenetic modification with the potential to promote genome instability, cancer growth, tumor angiogenesis, metastasis, and oncotherapy resistance. An increasing number of studies have identified the crucial carcinogenic role of histone Kla in HCC [100]. Increasing the intracellular concentrations of lactate in a dose-dependent manner can effectively enhance the level of Kla [101]. Up to now, the effects of lactylation are not well known but the increase of lactate in HBV infection may trigger of Kla activation. Disorders of signaling pathways play a critical role in HCC tumorigenesis. The Kla induced activation of Wnt/β-catenin, MAPK, NOTCH, MTOR signaling [100]. The dysregulation of these pathway by HBV can lead to liver damage and hepatocellular carcinoma HCC [102]. Hence, targeting histone Kla can possibly/potentially inhibit the activation of HCC-related pathways and consequently improve the overall prognosis.

It is generally acknowledged that M2 macrophages have a pro-tumor role, while M1 macrophages have anti-tumor effects [46]. Li et al. demonstrated that HBV might promote M2 polarization of macrophages to impede the Th1 cell immune response, leading to persistent infection and disease progression [103]. Another study found that hepatitis B core antigen (HBcAg) effectively impairs M2 polarization and produces inflammatory cytokines [104]. On the other hand, other researchers have found that in M1 macrophages, lactate stimulates gene transcription through Kla to promote homeostasis [105]. Based on the available findings, we can hypothesize that by increasing lactate, HBV leads to liver damage indirectly through Kla [106], and Kla is expected to be a novel therapeutic target for HCC.

### Lactate and autophagy

Autophagy is a cellular degradation and recycling process that plays a critical role in maintaining cellular homeostasis across all eukaryotes and also is highly conserved in them [107]. It selectively degrades viral particles or cellular components, which either facilitates or inhibits viral replication. Most viruses have evolved strategies to escape or exploit autophagy [108]. Increasing evidence has proved that HBV causes incomplete autophagy and manages to avoid being degraded by the autophagic process. Conversely, during CHB infection, HBV-induced autophagy worsens the condition of the infected liver and contributes to the pathogenesis of HBV-associated HCC [109]. In fact, HBV utilizes or hijacks the autophagy machinery with the purpose of replication [110].

As we mentioned above, HBV stimulates lactate production through anaerobic glycolysis. Lactate can maintain autophagy in cancer. In this situation, lactate dehydrogenase B (LDHB), catalyzing the conversion of lactate and NAD^+^ to pyruvate, NADH and H^+^, controls vesicle maturation, lysosomal acidification, and intracellular proteolysis in cancer cells [111]. Induction of autophagy in HCC may stimulate glycolysis to promote tumor progression. Autophagy induces nuclear translocation of β-catenin to increase the expression of MCT1, leading to induction of glycolysis in HCC [112]. To explore the relationship between autophagy and lactate, we hypothesized that HBV-induced lactate worsens the condition of the infected liver and contributes to the pathogenesis of HBV-associated HCC.

## Conclusion

A key characteristic of cancer cells that distinguishes them from normal cells is the increase in glycolysis with concomitant lactate production. CHB infection is a major global cause of HCC and in hepatocytes, HBV increases overall glucose consumption and lactate production. Lactate is generally immunosuppressive by reducing cytotoxicity, IFN production, antigen presentation, maturation, activation, and differentiation. Additionally, lactate can enhance apoptosis and histone lactylation to promote tumor progression. In the presence of lactate, the innate immune cells (MΦs, DCs, NK, and neutrophils) and adaptive immune cells (T cells) are dampened. Remarkably, lactate level is associated with increased mortality rates. This review aims to highlight the significance of our understanding of increased lactate levels in those with HBV infection, which can be helpful in risk assessment. Nonetheless, further studies are required on lactate levels in HBV infection and their effects on the immune system to identify potential therapeutic targets. Subsequent research will hopefully shed some light on the relative contribution of lactate versus lactate-derived metabolites, such as acetyl-CoA in the development of viruses-related cancers.

## Data Availability

No datasets were generated or analysed during the current study.

## Abbreviations

ADP: Adenosine diphosphate
ATP: Adenosine triphosphate
CCR7: C-C chemokine receptor type 7
CTLA4: Cytotoxic T-Lymphocyte Associated Protein 4
DCs: Dendritic cells
FADH2: Flavin adenine dinucleotide
FAS: Fatty acid synthesis
FOXP3: Forkhead box P3
FAK: Focal adhesion kinase
FIP 200: Family interacting protein of 200
HBV: Hepatitis B virus
GAPDH: Glyceraldehyde-3-phosphate
HCC: Hepatocellular carcinoma
HBx: Hepatitis B virus X protein
HBXIP: Hepatitis B virus X-interacting protein
IFN-γ: Interferon-gamma
IL: Interleukin
IFNs-I: Type I interferons
IKKε: IκB kinase-ε
LDH: Lactate dehydrogenase
LHBS: Large viral surface antigens
Kla: Histone lysine lactylation
MΦs: Macrophages
MAPK: Mitogen-activated protein kinase
MCTs: Monocarboxylate transporters
mTOR: Mammalian target of rapamycin
MAVS: Mitochondrial antiviral-signalling protein
NADH: Nicotinamide adenine dinucleotide
NO: Nitric oxide
NK: Natural killer
NKT: Natural killer T cells
NFAT: Nuclear factor of activated T cells
NLR: Neutrophil-to-lymphocyte ratio
NE: Neutrophil elastase
NF-κB: Nuclear factor-κB
OXPHOS: Oxidative phosphorylation
PKM2: Pyruvate kinase M2
PPP: Pentose phosphate pathway
PEP: Phosphoenolpyruvate
PLZF: Promyelocytic leukemia zinc-finger
PPARs: Peroxisome proliferator-activated receptors
RIG-I: Retinoic acid-inducible gene I
TCA: Tricarboxylic acid
TME: Tumor microenvironment
TNF-α: Tumor necrosis factor-α
TCR: T-cell receptor
Tregs: Regulatory T cells
Th1: T-helper 1
TNFRSF18: TNF Receptor Superfamily Member 18
IRF3: Interferon regulatory factor 3
TBK1: TANK-binding kinase 1
GPCR: G-protein coupled receptor
GB: Granzyme B
VEGF: Vascular endothelial growth factor
